# Patient Anxiety in Oro-Dental Procedures: A Retrospective Observational Study of Biopsychosocial Aspects

**DOI:** 10.3390/bs16010108

**Published:** 2026-01-13

**Authors:** Elena Gabriela Strete, Cristina Raluca Bodo, Dora-Mihaela Cîmpian, Mihaela Diana Corodan Comiati, Emese Lukacs, Mădălina-Gabriela Cincu, Ramona-Amina Popovici, Alexandra Enache, Sorina Enasion, Lorena Mihaela Grebenișan, Andreea Sălcudean

**Affiliations:** 1 Department of Psychiatry, George Emil Palade University of Medicine, Pharmacy, Science and Technology of Targu Mures, 540142 Targu Mures, Romania; elena.buicu@umfst.ro (E.G.S.); emese.lukacs@umfst.ro (E.L.); lorena-mihaela.muntean@umfst.ro (L.M.G.); 2 Department of Ethics and Social Sciences, George Emil Palade University of Medicine, Pharmacy, Science and Technology of Targu Mures, 540142 Targu Mures, Romania; cristina.bodo@umfst.ro (C.R.B.); cimpiandora@umfst.ro (D.-M.C.); andreea.salcudean@umfst.ro (A.S.); 3 Psychiatry Clinic 1, County Clinical Hospital Mures, 540136 Targu Mures, Romania; corodan.mihaela@yahoo.ro; 4 Doctoral School of Faculty of Medicine, George Emil Palade University of Medicine, Pharmacy, Science and Technology of Targu Mures, 540142 Targu Mures, Romania; 5 Department of Management and Communication in Dental Medicine, Faculty of Dental Medicine, Victor Babes University of Medicine and Pharmacy of Timisoara, 300041 Timisoara, Romania; 6 Discipline of Legal Medicine, Bioethics, Deontology and Medical Law, Victor Babes University of Medicine and Pharmacy of Timisoara, Eftimie Murgu Sq., 300041 Timisoara, Romania; enache.alexandra@umft.ro; 7 Doctoral School, Victor Babes University of Medicine and Pharmacy of Timisoara, Eftimie Murgu Sq., 300041 Timisoara, Romania; sorina.enasoni@umft.ro

**Keywords:** dental anxiety, fear, depression, somatoform disorders, oral health, health knowledge, attitudes, practice

## Abstract

Aim of the study: Anxiety about dental treatment is one of the main barriers to accessing dental services and, at the same time, a well-known problem for dentists. The main objective of this observational pilot study was to assess the prevalence and determinants of dental anxiety and severe forms compatible with dental phobia among adult dental patients and to explore their association with psychological distress, as well as patients’ preferences for methods to reduce pain and anxiety during dental treatment. Materials and Methods: We carried out a pilot observational study using two well-established questionnaires, namely the BSI-18 (Brief Symptom Inventory-18), which assesses the psychological distress of patients visiting the dentist, and the DAS (Dental Anxiety Scale), which evaluates dental anxiety toward dental treatment. The questionnaires were administered in independent dental practices in Timisoara, and the study was conducted between August 2024 and January 2025 on a sample of 231 persons. Results: The results of our study revealed a clear link between sources of oral health information, the high prevalence of anxiety as a personality trait, anxiety towards the dentist, and referral to dental services. Conclusions: The intensity of dental anxiety is higher before the therapeutic manoeuvre. It has been found, however, that people who are more educated experience lower levels of anxiety in specific situations.

## 1. Introduction

The phenomenon of dental anxiety is a complex interplay of biological, psychological, and social factors that influence the patient’s behaviour and attitude towards dental treatment. Dental anxiety is a prevalent problem that affects a substantial segment of the population, with studies indicating that over 80% of people experience some level of fear related to dental treatment and about 20% actively avoid dental care because of these fears (Silveira et al., 2021; Done et al., 2023).

Pain plays a major role in anxiety (Franke et al., 2010). According to Petersen’s findings, dental anxiety tends to emerge later in life, often becoming more pronounced in adulthood (Petersen et al., 1995). Danila et al. (2003) demonstrated that fear of pain and the intensity of dental anxiety are positively correlated. Previous studies have reported that traumatic experiences with the dentist, both in childhood and adolescence, are among the most common causes of patient anxiety when entering the dental practice (Balcos et al., 2021). An author has stated that traumatic experiences—especially in childhood—are frequent antecedents of dental fears and phobias. People with invasive experiences at the dentist remain more often anxious, retain a fear of pain, and have a negative attitude towards dentists, unlike people with non-invasive experiences (Pleşea Condratovici et al., 2018). In this context, it is important to distinguish between dental anxiety and dental phobia. Dental phobia represents the most severe form of dental fear and is typically classified as a specific phobia, characterized by intense fear, avoidance of dental treatment, and clinically significant impairment in daily functioning. Individuals with dental phobia frequently postpone or completely avoid dental visits despite recognized treatment needs. Previous research has shown that dental phobia is associated with traumatic dental experiences, higher psychological distress and comorbid anxiety symptoms, as well as poorer oral health outcomes due to avoidance of care (Armfield & Heaton, 2013; De Stefano, 2019). Biological factors, such as the physiological impact of anxiety on the body, play a significant role in exacerbating dental fear. For example, sympathetic stimulation can increase heart rate and produce physiological symptoms that increase a patient’s anxiety during dental procedures (Armfield & Heaton, 2013; Singh et al., 2014). Patients who experience higher levels of anxiety before dental treatment often perceive greater pain during procedures, indicating a psychological component intertwined with physical experiences of pain (Lin et al., 2017).

From a psychological perspective, past traumatic dental experiences are notable predictors of future dental anxiety, as patients who have undergone painful or stressful dental procedures often carry these negative associations with them into subsequent visits (Kumari et al., 2023). This association extends beyond the dental environment, suggesting that previous painful non-dental experiences, such as trauma, may amplify dental anxiety (Humphris & King, 2011).

Social factors also strongly influence dental anxiety. The role of parental anxiety, especially in pediatric populations, cannot be underestimated. Children whose parents have high levels of dental anxiety are more likely to have similar emotional responses when faced with dental treatment (Abbas et al., 2023). In addition, societal perceptions and stigma surrounding dental treatment contribute to people’s reluctance to seek medical care, further perpetuating poor oral health outcomes due to avoidance behaviors (Alansaari et al., 2023). The treatment process itself, including the behaviors and attitudes of dental professionals, can significantly influence patients’ anxiety, requiring a compassionate and understanding approach to care giving (Singh et al., 2014).

Effective management of dental anxiety is essential for increasing patient compliance and promoting positive treatment experiences. Non-pharmacological approaches, such as cognitive-behavioral therapy (CBT) and relaxation techniques, are effective in changing patients’ perceptions and experiences of dental treatment anxiety (Wide & Hakeberg, 2021; Pathak et al., 2024). Specific interventions such as multimedia distractions or music therapy have also been shown to alleviate anxiety through psychological engagement and environmental modulation (Pathak et al., 2024). In addition, the use of a regimen of systematic desensitization and positive reinforcement may help to reduce anticipatory anxiety, thereby improving overall treatment outcomes for patients with high anxiety (Sivaramakrishnan et al., 2022).

Effective management of dental anxiety is essential for increasing patient compliance and promoting positive treatment experiences. A stepped-care approach is usually recommended, combining non-pharmacological and pharmacological strategies according to the severity of anxiety and the presence of dental phobia. Non-pharmacological methods include effective communication and rapport building (e.g., tell-show-do, clear information, empathy), cognitive-behavioural techniques such as graded exposure and relaxation training, as well as distraction procedures (music, audiovisual media, virtual reality) and environmental modification of the dental office. These interventions aim to change patients’ cognitions and expectations and to reduce anticipatory anxiety (Armfield & Heaton, 2013; Singh et al., 2014; Wide & Hakeberg, 2021; Pathak et al., 2024).

For patients with marked dental phobia or when behavioural methods alone are insufficient, adjunctive pharmacological approaches such as oral or intravenous sedation, inhalation sedation with nitrous oxide, or, in selected cases, treatment under general anaesthesia may be indicated as part of a comprehensive anxiety-management plan. Integrating behavioural, communicational and pharmacological adjuncts is therefore essential for optimizing treatment outcomes in highly anxious or phobic dental patients (Armfield & Heaton, 2013; Wide & Hakeberg, 2021; Sivaramakrishnan et al., 2022).

The present study uses two standardized instruments, the Brief Symptom Inventory-18 (BSI-18) and the Dental Anxiety Scale (DAS). The BSI-18 is a short self-report questionnaire designed to assess psychological distress, evaluating somatization, depression, and anxiety. The DAS is one of the most widely used tools for measuring dental anxiety. The scale has been widely recognized for its validity and reliability. It remains one of the most utilized tools in dental practice to gauge patient anxiety before treatment. Previous empirical studies have reported good reliability and validity of these instruments and have also shown significant associations between dental anxiety, psychological distress, depression, somatization, and sociodemographic characteristics, supporting the relevance of investigating these variables together within a biopsychosocial framework (Sivaramakrishnan et al., 2022; Zinke et al., 2019).

There are still important gaps in the existing literature regarding dental anxiety. Most previous studies have focused either on dental fear alone or on general psychological distress, without simultaneously examining their interaction within a biopsychosocial model. In addition, many studies have been conducted in clinical subpopulations or small samples, limiting the generalizability of the results. There is also limited evidence regarding the relationship between dental anxiety, somatization, depression, anxiety symptoms, and patterns of dental service use in adult populations. The present study aims to address these gaps by simultaneously assessing dental anxiety and psychological distress using validated instruments (DAS and BSI-18) in a sample of adult dental patients, and by exploring the influence of sociodemographic factors. In doing so, this study contributes to a better understanding of the biopsychosocial determinants of dental anxiety and to improving strategies for patient management in dental practice.

In the context of the above, the present study aims to assess the prevalence and determinants of dental anxiety and severe dental fear compatible with dental phobia, as well as the therapeutic methods offered by dentists and preferred by patients to reduce pain and anxiety before and during dental treatment.

Based on the main objective and the observational study design, the following research questions were formulated:

1. What is the level of dental anxiety among adult dental patients? Are there differences according to gender and age groups (<46 versus ≥46 years)?
2. What are the main triggers of dental anxiety during dental treatment?
3. What methods to reduce pain and anxiety are currently offered by dentists, and which methods are preferred by patients?
4. Are patients willing to use anxiety-management methods in order to increase the frequency of dental visits?
5. Is dental anxiety associated with psychological distress (somatization, depression, and anxiety) measured using the BSI-18?

### Research Hypotheses

On the basis of the existing literature and the above research questions, the present exploratory pilot study was guided by the following hypotheses:

**H1.**  *Higher levels of psychological distress (somatization, depression, anxiety, and GSI scores on the BSI-18) are positively associated with higher levels of dental anxiety (DAS scores)*.

**H2.**  *Women and younger adults (<46 years) report higher dental anxiety and psychological distress than men and older adults (≥46 years)*.

**H3.**  *Patients who attend the dentist only when they experience pain show higher levels of dental anxiety than patients who attend the dentist regularly for routine check-ups*.

## 2. Materials and Methods

### 2.1. Study Design and Cohort Description

The present study is a retrospective–descriptive observational pilot study, which initially included 300 respondents (patients with dental problems, attending dental surgeries) who were asked to participate in the study. Participants were recruited through a non-probabilistic convenience sampling procedure. The sample consisted of adult patients attending independent dental practices during the study period who met the inclusion criteria and agreed to participate. Of the 300 participants, only 231 (77%) were included in the present research; 69 respondents were excluded because they either did not wish to complete the questionnaires or left questions incomplete. Final data were collected independently in several individual dental practices in Timisoara city (Romania) over 6 months (August 2024–January 2025).

**Figure 1:**
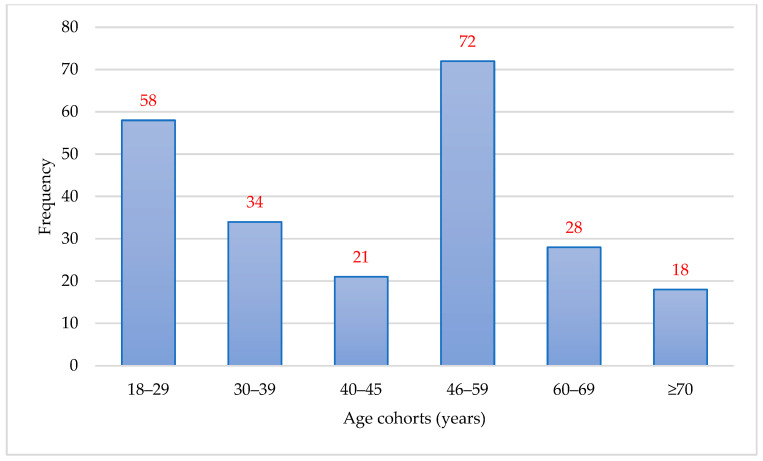
Frequency distribution of age cohorts in the study sample.

Figure 1 shows the frequency distribution of age cohorts in the study sample. The largest proportion of participants belonged to the 46–59 years age group, followed by the 18–29 and 30–39 years groups, while the smallest proportion was represented by participants aged 70 years and older.

### 2.2. Data Collection

Data were collected following the application of the BSI-18 and DAS questionnaires, which were given to the study participants when they presented themselves at the dental offices and asked to complete them before their routine treatments. The questions contained in the questionnaires also referred to the sociodemographics of the respondents, namely age, background, and gender. The mandatory condition for participation in the study was age; all participants had to be 18 years of age and were initially asked to complete written informed consent.

The Brief Symptom Inventory-18 (BSI-18) is an 18-item self-report questionnaire designed to assess psychological distress and associated psychiatric symptoms in various populations. Developed as a concise version of the Brief Symptom Inventory (BSI), which comprised 53 items, and ultimately derived from the Symptom Checklist-90-Revised (SCL-90-R), the BSI-18 aims to provide a quick yet effective screening tool for detecting symptoms of somatization, anxiety, and depression (Zinke et al., 2019). Each of the three subscales within the BSI-18 contains six items, allowing for a more focused assessment of the respondents’ experiences over the prior week, using a 5-point Likert scale ranging from 0 (not at all) to 4 (extremely) (Faure-Carvallo et al., 2024).

The Dental Anxiety Scale (DAS) is a prominent instrument used to assess dental anxiety among patients. Originally derived from Corah’s early work, the DAS consists of a limited number of questions that focus on anticipating dental treatment, allowing healthcare providers to measure anxiety levels effectively and reliably (Sharma et al., 2019; Shankar et al., 2024).

### 2.3. Statistical Data Analysis

Quantitative variables were summarized using means and standard deviations. For comparative analyses, age was dichotomized into <46 and ≥46 years based on the sample mean, and DAS scores were categorized into low, medium and high anxiety.

The collected questionnaires were entered into an Microsoft Excel (Microsoft Corp., Redmond, WA, USA) database and imported into IBM SPSS Statistics, version 20.0 (IBM Corp., Armonk, NY, USA) for analysis. In the present study, different statistical tests were used according to the type of data and research objective. Student’s *t*-test for independent samples was used to compare mean DAS and BSI-18 scores between two groups (e.g., gender and age categories). The Chi-square test was applied to examine associations between categorical variables such as anxiety level categories and sociodemographic characteristics. Pearson correlation coefficients were calculated to evaluate the relationship between dental anxiety (DAS) scores and psychological distress dimensions assessed by the BSI-18 (somatization, depression, anxiety, and GSI). p-values equal to or less than 0.05 were considered statistically significant. In addition, the Kruskal–Wallis test was used to compare non-normally distributed variables across more than two independent groups, specifically for the analysis of dental anxiety according to the frequency of dental visits.

In this study, the biopsychosocial aspects were operationalized as follows. The psychological component was assessed using the BSI-18, measuring anxiety, depression, somatization and the Global Severity Index. Dental anxiety was evaluated with the DAS. The biological/behavioural component was reflected by symptoms related to pain and physiological arousal reported in anxiety items. The social component included sociodemographic characteristics (age, gender, education), frequency of dental visits, access to dental care and sources of oral-health information.

Participation in the study was voluntary. All participants received written information about the purpose of the research and signed informed consent prior to completing the questionnaires. The questionnaires were anonymous, no personal identifiers were collected, and participants were assured of the confidentiality of their data. They were informed that they could withdraw from the study at any time without any consequences for their dental treatment.

Based on the BSI-18 Global Severity Index (GSI), participants were classified into three psychological distress groups: low, moderate and high distress. Dental anxiety (DAS scores) was compared across these three groups using the Kruskal–Wallis test.

For BSI-18 subscales (depression, anxiety, somatization), participants were classified into low versus high symptom groups, and mean DAS scores were compared using independent- samples *t*-tests or Mann–Whitney U tests when normality assumptions were not met.

Ethical considerations: The study was conducted in accordance with the ethical standards of the Declaration of Helsinki. Ethical approval was obtained from the Ethics Committee of the “Victor Babeș” University of Medicine and Pharmacy, Timișoara (No.: 89/2 June 2024, rev. 2025).

## 3. Results

In the total sample, 161 patients (69.7%) were classified as having low dental anxiety, 59 patients (25.5%) showed moderate dental anxiety, and 11 patients (4.8%) had high dental anxiety according to the DAS categories. The high anxiety group can be regarded as representing severe dental anxiety or probable dental phobia. Within this group, 1 man (1.6% of male participants) and 10 women (6.0% of female participants) were identified as having high dental anxiety. These distributions are also presented in Table 1. The DAS results from the present study show that, from question 7 onwards, the tendency to select an answer with a higher anxiety level increased. This can be explained by the fact that these questions refer to dealing with situations that cause dental anxiety. Concerning these challenging situations, the study by Pănăzan et al. (2023) concludes that both patients with low anxiety and those with higher anxiety fear tooth preparation and extraction the most. It thus becomes evident that the main triggers of the anxiety reaction are closely linked to the treatment itself, i.e., the active action on the patient’s teeth.

In the present study, the fear of pain seems to play a very important role in the development and triggering of anxiety both from the point of view of patients and physicians. In a study by Hajj et al. (2021), the authors reported that in 58% (*n* = 137) of patients, they were afraid or had an unpleasant feeling when they heard the sound of the turbine. Approximately 42% of the patients reported this feeling during the injection of the local anesthetic (i.e., “stinging”), and 40% experienced it at the mere sight of the syringe. Other researchers have reported, in descending order, pain, injection, and turbine as anxiety-triggering stimuli. In addition to these, unknown and unpredictable situations during treatment are a possible cause for the development and triggering of dental anxiety (Hare et al., 2018). These results from the literature coincide with the results of the present study. Pain, fear of the unknown, injection, and turbine noise were reported as trigger stimuli in descending order. Thus, it is assumed that there are certain anxiety-provoking situations in dental practice. Despite the development of modern methods of anaesthesia in dentistry, pain is still the most common triggering stimulus.

Table 1 summarizes the levels of dental anxiety according to the specific triggering situations. The highest percentages of “very anxious” and “extremely anxious” responses were reported for situations involving injections, turbine noise, and tooth extraction, while routine situations (e.g., waiting room, dental cleaning) were predominantly associated with low anxiety levels.

### 3.1. Regular Visits to the Dentist

**Figure 2:**
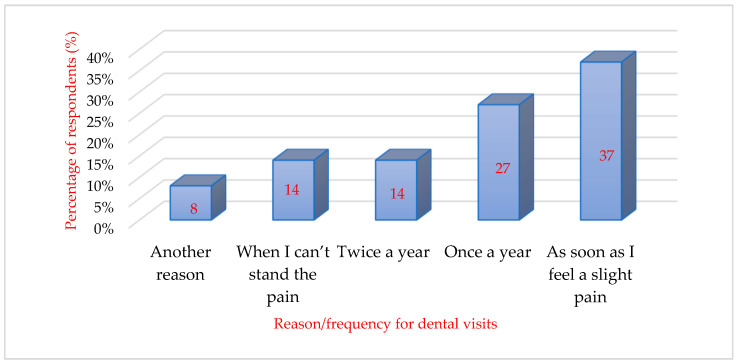
Patients’ referral rate to the dentist.

When asked about the regularity of visits to the dentist’s office, it can be seen that about one-third of the respondents (*n* = 85) go to the dentist only when they experience slight pain. About a quarter of the respondents (*n* = 62) go to the dentist regularly, about once a year, and 33 respondents out of 231 participants reported a visit to the dentist only when the pain had already become unbearable. The resulting data are shown in Figure 2.

The sum score of the DAS scale was compared with the regularity of dental visits. Participants were divided into five groups according to their self-reported frequency of dental visits, a criterion commonly used in epidemiological studies on dental service utilization (Alansaari et al., 2023):

(a) Patients who go regularly to the dentist even if they do not feel pain (twice a year);
(b) Patients who regularly visit the dentist (once a year);
(c) Patients who go to the dentist as soon as they feel pain;
(d) Patients who go to the dentist when they cannot stand the pain anymore;
(e) Other categories of patients.

After applying the Kruskal–Wallis statistical test, a statistically significant difference (*p* = 0.002) was observed between the patient groups considered. The first 3 groups were less worried (less anxious) than the last 2 groups.

### 3.2. Methods to Reduce Pain and Anxiety Identified by Patients

**Figure 3:**
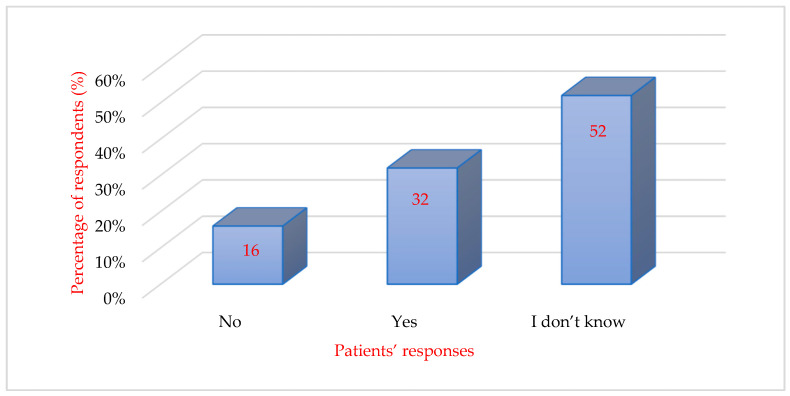
Perception of the dentist’s involvement in reducing patient anxiety.

When asked if the dentist offers ways to reduce anxiety, pain, and stress during treatment, 32% (*n* = 73) of the participants answered yes, and half of the respondents (*n* = 120) did not know if the dentist offers such methods. Only 38 respondents denied this. The data analyzed are presented schematically in Figure 3.

**Figure 4:**
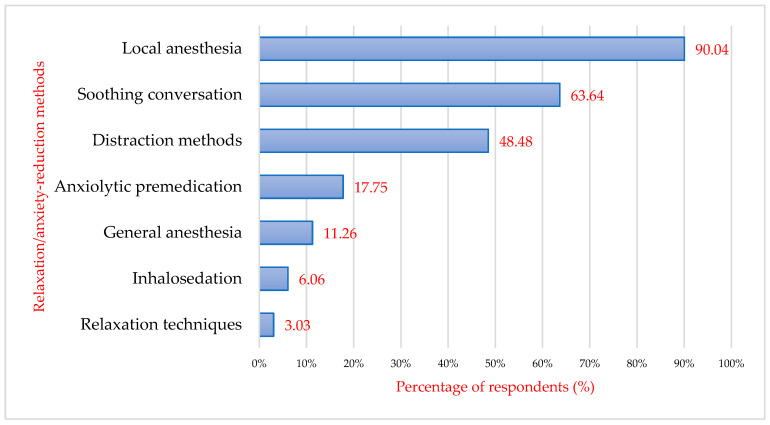
Patients’ perception of relaxation techniques used in the dental office by doctors to reduce anxiety (multiple response).

Also in terms of reducing the degree of anxiety, when asked about patients’ perception of the concrete methods used in the dental office by dentists regarding the treatment to be performed, about 208 of the respondents (90.04%) answered “local anesthesia” followed by “soothing conversations” (63.64%, *n* = 143). Distraction methods were ticked by 112 respondents (48.48%), 41 participants (17.75%) were sedated with anxiolytic premedication, and 14 respondents (6.6%) answered that they were sedated with a gas inhalation pin. In a proportion of respondents (11.26%, *n* = 26), general anaesthesia was performed (Figure 4). Hypnosis and psychotherapy were not indicated in this study by any patient, as these methods of anxiety reduction require specialized personnel. In the dental practices included in the present study, specialist psychotherapeutic personnel were not available, and such interventions were not routinely offered to patients.

**Figure 5:**
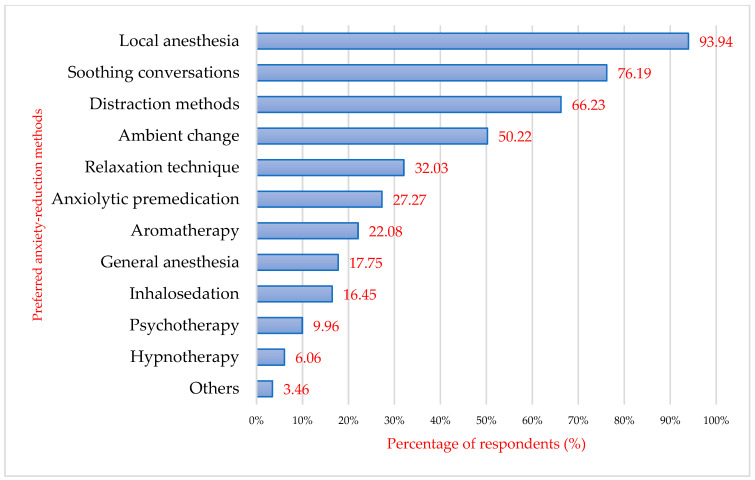
Methods to reduce anxiety desired by patients (multiple response).

Patients were then asked to specify which of the methods mentioned would make their dental treatment and, therefore, their visit to the dentist easier. Local anaesthesia ranked first (93.94%, *n* = 217), followed by soothing conversations (76.19%, *n* = 176), and then distraction methods (e.g., audio-video through music or videos) (66.23%, *n* = 153). Changing the environment was desired by 116 patients interviewed (50.22%). One-fifth of patients would appreciate aromatherapy in the dental office for relaxation (*n* = 63). Inhalosedation is desired by only 16.45% of patients (*n* = 38). Hypnotherapy is appreciated by 6.06% of respondents, while general anaesthesia is appreciated by 17.75% (*n* = 41) and psychotherapy by 9.96% (*n* = 23) (Figure 5). Psychotherapy and aromatherapy are gaining more and more ground, as patients have started to take more care of their mental health and are opening up to more and more alternative methods of treatment such as psychotherapy, homeopathic medicine, herbal medicine, aromatherapy, etc.

Patients were asked to indicate which methods would make dental treatment easier, and they were allowed to select more than one option (multiple responses). Table 2 presents the distribution of preferences according to dental anxiety categories (low, moderate, high/phobia). There is a clear gradient according to the severity of dental anxiety: local anaesthesia is the most preferred method in all groups and is almost universal in high anxiety/phobia patients (11/11). Patients with low anxiety mainly choose communication-based strategies (soothing conversation 95; distraction 70). Patients with moderate anxiety increasingly choose both communication and pharmacological options (general anaesthesia 20; inhalosedation 15). High anxiety/dental phobia patients prefer advanced methods (general anaesthesia 11; anxiolysis/sedation 11; psychotherapy/hypnotherapy 8–11). Overall, preferences progress from simple non-pharmacological strategies in low fear toward sedation and psychological interventions in high fear/phobia, which supports tailoring management according to anxiety severity.

When patients were asked if they would like to receive more information about their dentist suggesting alternative methods for relieving pain and reducing anxiety, almost all respondents said yes (*n* = 206).

Reliable pain relief and thus a possible reduction in anxiety can be achieved by local anaesthesia (Gomes et al., 2020; Peres et al., 2018; Kisely et al., 2016; Derogatis, 2001; Franke et al., 2010), and positive affirmations, empathy, and patience can show the patient that his problems are taken seriously and the dentist is doing his best to understand him.

In this study, the performance of local anesthesia, soothing conversations, distraction methods, an appropriate atmosphere, and ambience are desired in descending order by patients. In addition, since 66% of patients indicated distraction methods as helpful, technology-based tools may also be relevant. Modern audiovisual approaches, such as virtual reality or augmented reality, can divert attention from dental stimuli and reduce situational anxiety, especially in highly anxious patients. These methods may represent a useful complement to traditional reassurance and communication strategies.

**Figure 6:**
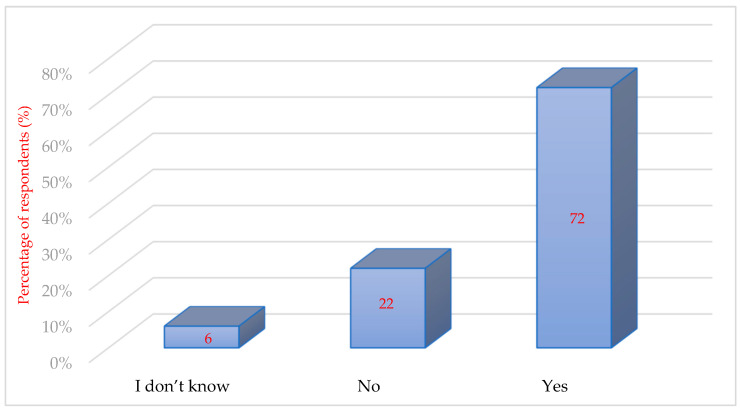
Patients’ expression of increased frequency of referral to the dentist if more anxiety-reducing techniques were applied and they no longer felt pain.

Although it can be assumed that only behavioural psychotherapy can treat the causes of anxiety, it has been approached by only a small percentage of patients. In comparison, patients more frequently reported a willingness to be treated with anxiolytic premedication than with psychotherapy, which is probably based on the patient’s knowledge of the issue. Many patients think that with proper premedication, they can suppress their fears without the need for treatment of their root causes (Shenoy & Sequeira, 2010). Moreover, the respondents expressed their opinion about the increased frequency of going to the dentist if more techniques were applied to reduce anxiety and if they did not feel pain. The majority of respondents answered yes (72%, *n* = 166), 51 respondents answered no (22%), and 14 participants (6%) could not decide (Figure 6).

### 3.3. Comparison of Patient Age, Dental Anxiety, and Psychological Distress

To analyse whether the age of the respondents has any relationship with dental anxiety and psychological distress, the respondents were divided into 2 categories: young patients < 46 years of age (113/231) and those ≥46 years of age (118/231). The age of 46 was chosen because it represents the average age of our sample. This data was then compared with the DAS scale, and the patient was placed according to their score on the questionnaire (Table 3).

Results showed that the majority of patients in both age groups were placed in the low anxiety category. In all categories, the spread of the two age groups was similar. There were more patients under the age of 46 in the high anxiety category than those aged 46 and over. There were slightly more patients aged 46 and over in the low anxiety age group than those under 46. Therefore, in terms of anxiety as a function of patient age, no statistical significance was found in the data analysed.

The same age groups were then divided into the BSI-18 categories of Somatization, Depression, and Anxiety, and the Global Severity Index was calculated. Patients younger than 46 years of age had about the same score as patients aged 46 years or older in the Somatization category. In the Depression category, younger patients had a higher average than older patients. This was found to be statistically significant. Also statistically significant was anxiety, which was found to be higher in younger patients than in older patients. The overall severity index was higher for younger patients than for older patients. Therefore, the findings indicated a higher presence of depression and anxiety in younger patients than in older patients. Results showed significantly higher levels of depression and anxiety in younger patients compared with older patients, while somatization scores were similar between groups. These differences may reflect age-related variation in exposure to stressful experiences and coping patterns, as also reported in previous studies (Guentsch et al., 2017; Bodo et al., 2024; Lenk et al., 2013; Salcudean & Lica, 2024; Sălcudean et al., 2024b; Sălcudean et al., 2023). Screening instruments such as the DAS may therefore be useful for identifying patients with higher dental anxiety levels in clinical settings. Comparing DAS categories with patient age showed no statistical significance. These findings were in stark contrast to those of similar studies reported in the literature (Anfield, 2010; De Stefano, 2019; Wilkinson, 2013; Franke et al., 2011), showing a decrease in the prevalence of dental anxiety in older populations compared to younger populations. One explanation could be the classification into only two age groups and a generalization of the words ‘young’ and ‘old’. A shortcoming of DAS is the non-inclusion of local anaesthetics and a strong influence of the patient’s judgment on the treatment. Patients are most afraid of pain and injury from the injection (Corah, 1969; Herlo et al., 2024; Kalinovic et al., 2024), even though 40% of younger age groups prefer treatment with anaesthesia (Fayad et al., 2017).

### 3.4. Comparison of Gender, Dental Anxiety, and Psychological Distress

We looked for statistical differences in DAS between female and male patients (Table 3). Most patients are classified as having low dental anxiety. Patients with high dental treatment anxiety were in the minority. A statistically significant difference was found.

Table 4 shows the mean scores for male and female patients. It is observed that female patients had higher scores in the BSI-18 Somatization, Depression, Anxiety, and GSI categories. The DAS score results can also be defined as statistically significant. Female patients scored higher than male patients.

Female patients showed higher mean scores for Somatization, Depression, Anxiety, and the Global Severity Index (BSI-18) compared with male patients. Dental anxiety levels (DAS) were also significantly higher among women. These findings indicate that female participants were more vulnerable to psychological distress and dental anxiety than male participants.

When comparing dental anxiety and GSI in terms of patients’ gender, it was observed that female patients were more susceptible to psychological distress regarding dental treatment than male patients. Our results confirm previous studies (Hardy et al., 2018). Women are more likely to develop dental anxiety than men, as expected (Chaudhary & Ahmad, 2021; Marian et al., 2025). One explanation for this result could be higher levels of neuroticism in women than in men, which is correlated with anxiety (Cademartori et al., 2018; Hosoba et al., 2001). Therefore, we can say that females are more anxious than males.

### 3.5. Comparison of the Relationship Between Psychological Distress (BSI-18) and Dental Anxiety (DAS)

To compare the relationship between psychological distress and dental anxiety, a Pearson correlation was applied (Table 5). The results showed significant positive correlations between all BSI-18 categories and the DAS questionnaire. A correlation coefficient of r = 0.338 represents a moderate positive correlation, indicating that increases in psychological distress are moderately associated with increases in dental anxiety. A coefficient of r = 0.159 represents a weak positive correlation, showing a small but meaningful association between these variables.

### 3.6. Dental Anxiety According to Three Levels of Psychological Distress (BSI-18)

Based on the BSI-18 Global Severity Index, three distress groups were identified (Table 6): low distress (*n* = 134; 58.0%), moderate distress (*n* = 67; 29.0%) and high distress/high anxiety–fear (*n* = 30; 13.0%). Dental anxiety differed significantly across these three groups. Patients in the high-distress group reported the highest DAS scores (Mean = 14.9 ± 3.8), followed by the moderate-distress group (Mean = 10.6 ± 3.1) and the low-distress group (Mean = 7.8 ± 2.4). The overall difference was statistically significant (Kruskal–Wallis, *p* < 0.001). These findings indicate a dose–response relationship, with increasing levels of psychological distress being associated with progressively higher levels of dental anxiety and probable dental phobia. Only a small proportion of participants fell into the high-distress/high dental fear–phobia group (13%), which is consistent with the notion that true dental phobia is relatively rare in the general dental population.

### 3.7. Dental Anxiety (DAS) in Highly Depressive, Anxious and Somatization

Patients with high depressive symptoms on the BSI-18 reported significantly higher dental anxiety compared with those with low depressive symptoms (Table 7; Mean DAS 13.7 versus 8.9; *p* < 0.001). Participants with high BSI-18 anxiety scores also showed higher dental anxiety (Mean DAS 14.2 versus 9.1; *p* < 0.001). High somatization was associated with elevated dental anxiety (Mean 12.9 versus 8.7; *p* < 0.001). These findings indicate that patients with high levels of depression, anxiety, or somatization exhibit substantially higher dental anxiety.

## 4. Discussion

The present study showed that dental anxiety was significantly associated with psychological distress, including depression and anxiety symptoms. Younger patients and women reported higher levels of psychological distress, and irregular dental attendance was related to higher dental anxiety. Strong positive correlations were found between DAS and all BSI-18 dimensions.

In this study, we were concerned with fear and anxiety disorders, looking for similarities and differences between them, analysing the mechanisms of their formation, both from the perspective of psychology and from the point of view of the impact of postmodern society on the individual. Fear and anxiety have been differentiated in concrete situations from anxiety as a stable personality trait, which implies a psychological vulnerability to the disorders of this class.

Therefore, a study was conducted on the biopsychosocial factors influencing anxiety related to dental treatment and the dental practice and manifesting as barriers to accessing health services.

### 4.1. Dental Anxiety According to Psychological Distress

The correlations between psychological distress and dental anxiety could be an indicator that patients with a generally higher level of psychological symptoms (depression, somatization, anxiety) are at higher risk for developing specific anxieties, such as dental fear. A similar study comparing 212 patients with psychosomatic services with 95 healthy controls confirmed these results (Carter et al., 2014). These associations between psychological distress and dental anxiety are consistent with broader evidence showing that stress, emotional symptoms and somatic vulnerability interact across different populations. Studies conducted during the COVID-19 pandemic and beyond have demonstrated strong links between stress, inflammatory processes, depressive symptoms and behavioural disturbances (Tilinca et al., 2021; Sălcudean et al., 2023; Strete et al., 2025).

Vulnerability in specific groups, such as older adults facing unrecognized psychological distress, further supports the need to consider psychosocial factors in clinical assessment (Buicu et al., 2017). Additional research highlights how metabolic imbalance, depressive biomarkers and chronic stress responses may contribute to heightened emotional reactivity in medical contexts (Tilinca et al., 2023; Sălcudean et al., 2024b). Within dentistry, a recent review underscores that dental anxiety is a psychosocial factor with a major impact on quality of life and treatment avoidance (Sălcudean et al., 2024c). Similar findings in pregnant women experiencing high stress reinforce the importance of screening psychological burden in routine clinical care (Răchită et al., 2022). The large sample size in this study was favourable compared to similar studies. Also, the use of a general population and not just patients with an existing diagnosis of anxiety allowed an unbiased view of the results. However, only patients who voluntarily visited a dentist were interviewed. Patients with diagnosable dental anxiety and an aversion to dental visits could not be examined (Kisely et al., 2016; Silveira et al., 2021; Zinke et al., 2019).

Although our study did not directly diagnose dental phobia, some patients reported high dental anxiety combined with avoidance of dental care, a pattern frequently described in individuals with dental phobia (Armfield & Heaton, 2013). These findings highlight the importance of early identification of patients at risk of developing dental phobia and the implementation of preventive psychological and behavioural interventions in dental practice.

Therefore, recognizing the biopsychosocial aspects of anxiety in dental treatment has important implications for patient care. Dental professionals should prioritize open and honest communication with patients, allowing them to express their concerns and fears. Explain procedures in detail and ensure that they can help alleviate anxiety, reduce patients’ fear, and restore their confidence. Implementing behavioural techniques, such as relaxation exercises and distraction, can help patients manage their anxiety during treatment. For people with severe dental anxiety, pharmacological approaches such as anti-anxiety medication or sedation may be considered. Furthermore, incorporating cognitive behavioural therapy techniques into treatment plans can help patients identify and modify negative thoughts and behaviours associated with dental treatment (Hare et al., 2018; Singh et al., 2014; Pathak et al., 2024).

Implications for practice are awareness of the importance of adverse psychosocial conditions and psychological characteristics such as self-esteem, shame and isolation tendencies, and emotional lability in dental health, for example, in avoidance of dental treatment and lower compliance. Dentists should focus on people with psychosocial disadvantages and poorer psychological resources to improve treatment adherence (Cademartori et al., 2018; Chaudhary & Ahmad, 2021). Similar associations between psychological factors and treatment adherence have also been demonstrated in Romanian cardiovascular patients (Sălcudean et al., 2024a).

In addition to patient-related biopsychosocial factors, dentist-related characteristics such as gender, age, clinical experience and interpersonal communication style may also influence patients’ level of dental fear, perceived safety and trust in treatment. These provider-related variables were not assessed in the present study and should be included in future research to better clarify their role in the development and maintenance of dental anxiety and dental phobia (Humphris & King, 2011; Lin et al., 2017).

### 4.2. Dental Anxiety According to Age and Gender

This result could be explained by the fact that regular visits to the dentist lead to a decrease in anxiety related to dental treatment.

Dental anxiety patients are restricted in their daily routines. More often than not, these patients will only choose to make a dental appointment if the pain becomes too unbearable. If the dentist is unaware of the patient’s anxiety, the appointment may deteriorate. This should not be a person’s first experience of being afraid of dental treatment. Screening with DAS is quick and easy and can prepare the dentist in giving the best treatment to the patient. This would, however, require the dentist to be professionally trained to treat a patient who fears the environment. With the help of specialized doctors and a fully trained dentist, it is possible to reduce dental anxiety in susceptible individuals (Alansaari et al., 2023; Fayad et al., 2017).

### 4.3. Dental Anxiety and Dental Attendance Patterns

The main source of information for the patients who participated in the study was the mass media, followed immediately by the dentist’s recommendations. This is why we believe that the involvement of professionals is necessary to better control the quality of the message disseminated through the media. Information from talking to non-specialists is preferred by retired people, unskilled and skilled workers, the unemployed, and people with a secondary education. Patients who said that they get their information directly from the dentist are mostly high school, post-secondary, undergraduate, and postgraduate educated. Beyond traditional mass media, digital communication channels also play an increasingly important role; for instance, communication via Instagram responses was shown to effectively reduce dental anxiety in a randomized trial using social media platforms (Sivrikaya et al., 2021). Online communication tools, including mobile dental m-health applications, can provide accessible and personalized oral-health information, helping patients better understand dental procedures and what to expect during treatment. These tools may contribute to reducing dental anxiety by increasing familiarity with the dental environment, promoting preventive behaviours, encouraging regular dental visits, and strengthening the trust-based relationship between patients and their dentists.

In these circumstances, the results showed significances, further studies are needed to differentiate the types of media used by patients for information purposes according to their age. We believe that it is also useful to involve the management of press groups in health promotion programs aimed at different target groups, according to their specific needs and financial possibilities.

The data of the present study are consistent with the research hypotheses formulated in the Introduction, showing that individuals with higher psychological distress report higher levels of dental anxiety in specific dental situations. Identifying these individuals before treatment begins and lowering anxiety levels can help increase patient compliance and therapeutic success. In this regard, we believe that it is necessary to specialize the teachers in the Department of Oral Health and Behavioral Sciences in the field of cognitive-behavioural psychology or to collaborate with psychotherapists in practical work so that future doctors can learn some techniques for managing anxiety. An important aspect that deserves further emphasis concerns the developmental continuity of dental anxiety across the lifespan (Carter et al., 2014; Humphris & King, 2011; De Stefano, 2019). Previous studies have shown that negative or painful dental experiences during childhood and adolescence are among the most frequent antecedents of dental anxiety in adulthood. Anxiety established early in life may persist over time, influencing patterns of avoidance, irregular attendance, and heightened anticipatory fear later in adulthood. Furthermore, anxious adult patients may unintentionally transmit fearful attitudes toward dental care to their children through modelling and family communication, thereby contributing to an intergenerational cycle of anxiety and avoidance. These considerations highlight the importance of early preventive strategies, including child-friendly dental environments, adequate pain control, and psychological support aimed at reducing anxiety during paediatric dental care.

### 4.4. Limitations

This study has several limitations that should be acknowledged. To minimize selection, information and social desirability bias, participation was voluntary, questionnaires were anonymous, and were completed before dental treatment.

Information about the dentists’ characteristics (e.g., age, gender, years of experience or previous providers) was not collected in this study. Also, information on the regularity of dental visits was self-reported by the participants and was not verified against dental records or objective clinical data.

First, its cross-sectional and retrospective design does not allow causal inferences. Second, the data were based on self-report questionnaires, which may be influenced by recall bias and social desirability. Third, the sample was obtained through convenience sampling from dental practices; therefore, individuals who completely avoid dental treatment were not included, which may underestimate the true prevalence of severe dental anxiety or dental phobia. Finally, the study was conducted in a single geographical region, which may limit the generalizability of the findings; however, the relatively large sample size strengthens the validity of the observed associations.

Another limitation is that no data were collected regarding the characteristics of the dentists who previously treated the participants (e.g., age, gender, years of professional experience, type of provider, communication style). Therefore, we could not analyse whether specific provider-related factors influenced the development of dental fear and dental phobia or patients’ attendance patterns. Also, another limitation is that data regarding the frequency of dental visits were based solely on self-report and were not cross-checked with objective clinical records. Therefore, recall bias and over- or underestimation of dental attendance cannot be excluded.

## 5. Conclusions

Based on the obtained results, we can conclude that the intensity of dental anxiety is higher before the therapeutic manoeuvre. More educated people experience lower levels of anxiety in specific situations. We assume that making the waiting room environment as pleasant as possible, distracting the patient’s attention, and shortening the time the patient waits for the therapeutic procedure to be performed can help to decrease the level of dental anxiety. Study results also showed strong associations between dental anxiety and psychological distress (somatization, depression, anxiety). Anxiety in dental treatment is a complex phenomenon influenced by various biopsychosocial factors. The most important factors that cause fear in patients are pain, followed by unpleasant and traumatic experiences in the past, and uncertainty about what will happen.

Therefore, by fostering a supportive environment, promoting effective communication, and using appropriate strategies, dental professionals can help alleviate anxiety and improve the overall dental experience for their patients. These findings are consistent with our hypotheses and support the biopsychosocial model of dental anxiety.

## Figures and Tables

**Table 1:** Level of anxiety experienced by patients according to the trigger factor.

Item	Relax (%)	A Bit Anxious (%)	Medium Anxious (%)	Very Anxious (%)	Extremely Anxious (%)
How do you feel about you have to go to the dentist tomorrow?	77.3	12.6	3.3	2	4.8
You are in the waiting room, waiting to be called. How do you feel?	60	12.6	18.6	2	6.8
Imagine walking into the treatment room and smelling a typical dental practice. How do you feel?	58.6	16.6	15.5	3.3	6
You have taken a seat in the dental chair, and the doctor comes in. How do you feel?	71.4	9.3	9.3	6	4
Together, you look at the x-ray and discuss what needs to be done. How do you feel?	78.7	6	8.7	2	4.6
How do you feel when you are told that you need to have a dental cleaning?	71.4	8.6	9.3	4	6.7
Your dentist tells you that you have a cavity and it needs to be treated immediately. How do you feel?	56.7	6	24.7	5.3	7.3
The dentist changes the chair position and prepares the syringe. How do you feel?	44.6	4.7	26	10	14.7
Imagine you hear the typical turbine whistle. How do you feel?	46	12	24.6	7.4	10
Your dentist explains that the carious lesion is too deep and the tooth needs to be extracted. How do you feel?	0.7	4.7	64.7	11.3	18.6
A wisdom tooth has to be extracted, anesthesia has already been given, and the dentist takes the pliers in hand. How do you feel?	0.7	6.6	70	10	12.7

**Table 2:** Preferred anxiety- and pain-reduction methods stratified by dental anxiety level (DAS categories).

Method	Low Anxiety (n)	Moderate Anxiety (n)	High/Phobia (n)
Local anaesthesia	145	61	11
Soothing conversation	95	60	21
Distraction methods	70	55	28
Ambient change	60	40	16
Aromatherapy	30	22	11
Inhalosedation	12	15	11
General anaesthesia	10	20	11
Psychotherapy	5	10	8
Hypnotherapy	2	4	8

**Table 3:** Age of patients compared with dental anxiety (DAS) and psychological distress (BSI-18) categories.

**Age**			
	**<46 Years, n (%)**	**≥46 Years, n (%)**	**χ^2^ Test**
Anxiety	113 (49)	118 (51)	χ^2^ = 0.95 *p* = 0.62
Low	78 (69)	84 (71)	
Medium	30 (26)	29 (24)	
High	5 (5)	5 (5)	
**Psychological stress**	**Mean ± SD**		*t* **-Student test**
Somatization	1.91 ± 2.74	1.97 ± 3.01	*p* = 0.082
Depression	2.34 ± 4.47	1.43 ± 3.19	*p* < 0.0001
Anxiety	3.18 ± 3.33	2.42 ± 3.74	*p* < 0.00001
GSI	7.41 ± 7.88	5.74 ± 8.41	*p* < 0.0001

Note: GSI = Global Severity Index.

**Table 4:** Gender of patients compared with categories of dental anxiety (DAS) and psychological distress (BSI-18).

**Gender**			
	**Male, n (%)**	**Female, n (%)**	**χ^2^ Test**
Anxiety	63 (49)	168 (51)	χ^2^ = 14.9 *p* = 0.001
Low	48 (75)	113 (67)	
Medium	14 (22)	45 (27)	
High	1 (3)	10 (6)	
**Psychological stress**	**Mean ± SD**		*t* **-Student test**
Somatization	1.74 ± 2.71	2.12 ± 3.03	*p* = 0.014
Depression	1.69 ± 3.46	2.04 ± 3.23	*p* = 0.052
Anxiety	2.29 ± 3.05	3.18 ± 3.39	*p* < 0.00001
GSI	5.68 ± 7.96	7.32 ± 8.31	*p* < 0.0001

Note: GSI = Global Severity Index.

**Table 5:** Correlation between dental anxiety (DAS) and psychological distress (BSI-18).

	DAS Pearson Correlation	Two-Tailed *p*-Value	*n*
Somatization	0.256	*p* < 0.0001	228
Depression	0.159	*p* < 0.0001	227
Anxiety	0.338	*p* < 0.0001	228
GSI	0.302	*p* < 0.0001	229

**Table 6:** Dental anxiety (DAS) according to BSI-18 distress levels.

BSI-18 Distress Level	*n*	DAS Mean	SD
Low distress	134 (58)	7.8	2.4
Moderate distress	67 (29)	10.6	3.1
High distress	30 (13)	14.9	3.8
Total	231 (100)		

**Table 7:** Mean DAS scores according to BSI-18 symptom severity.

BSI-18 Dimension	Group	Mean DAS	*p*-Value
Depression	Low	8.9	
	High	13.7	<0.001
Anxiety	Low	9.1	
	High	14.2	<0.001
Somatization	Low	8.7	
	High	12.9	<0.001
Global Severity Index	Low	8.5	
	High	14.6	<0.001

Note: DAS scores compared using independent-samples *t*-test or Mann–Whitney U test where assumptions were not met.

## Data Availability

All the raw data presented in this study can be provided upon request by the corresponding author.

## Abbreviations

The following abbreviations are used in this manuscript:

BSI-18	Brief Symptom Inventory—18 items
DAS	Dental Anxiety Scale
SD	Standard Deviation
GSI	Global Severity Index
